# Self-propulsion of a grain-filled dimer in a vertically vibrated channel

**DOI:** 10.1038/s41598-017-14299-8

**Published:** 2017-10-27

**Authors:** C. Xu, N. Zheng, L.-P. Wang, L.-S. Li, Q.-F. Shi, Zhiyue Lu

**Affiliations:** 1 School of Physics, Beijing Institute of Technology, Beijing, 100081 China; 2 Key Laboratory of Cluster Science of Ministry of Education, Beijing, 100081 China; 3 Science and Technology on Electromagnetic Scattering Laboratory, Beijing, 100854 China; 4 James Franck Institute, University of Chicago, Chicago, 60637 USA

## Abstract

Steady dissipation of energy is a crucial property that distinguishes active particles from Brownian particles. However, it is not straightforward to explicitly model the dissipative property of existing active particles driven by a vibrating plate. We present a novel active particle that can be explicitly modeled by Newtonian dynamics of a conservative force field plus two asymmetrical dissipative terms. The particle is a dimer consisting of two ping-pong balls connected by a rigid rod, and its two balls are filled with granular particles of the same total mass but of different grain size. This dimer placed on a vibrating plate exhibits 3 types of motion – by tuning the frequency and the amplitude of the vibration, the dimer undergoes either a directed motion toward the small (or large) grain-filled side or an unbiased random motion. We investigate the various modes of motion both experimentally and numerically and show that the directed motion is a result of the asymmetric damping due to the size difference in the filling grains. Furthermore, the numerical simulation reveals that the dimer’s dynamics in either directed motion mode resembles a limit cycle attractor that is independent of its initial condition.

## Introduction

The broken time reversal symmetry by the dissipation of energy distinguishes active matter from a system of Brownian motion^1^. Important examples of active matter are constituted by natural and man-made objects capable of self-propulsion, including bacteria, spermatozoa, living animals, and Janus catalytic colloids. In recent years, active matter has attracted extensive attention from various fields due to the myriad interesting and counterintuitive behaviors^2,3^. Such behaviors include order-disorder transition^4^, giant number density fluctuation^5^, self-sustained density oscillation^6^, exotic pattern formation^7^ and chiral structures at macroscopic scale^8^.

Vibration-induced self-propelled granular particles provide an alternative example for active matter. With better controllability, they serve as an ideal experimental toolset to mimic microscopic forms of active matters, and potentially provide illuminating thoughts to explore universal physics at different length scales. Spontaneous collective motion^9–11^, such as persistent swirling, heterogeneous clustering, and orientational order in a monolayer of vertically vibrated granular rods has been intensively investigated to understand the bacterial behavior by analogy^12–15^. In contrast, much less research is concerned with the individual behavior of a self-propelled particle except for few researches^16–18^. Enhanced knowledge of the properties for individual particles will be potentially beneficial to the development of collective behavior of active particles.

Here we report a novel design of a tunable active dimer – two grain-filled ping-pong balls firmly connected by a rigid rod – that is able to perform horizontal self-propulsion on a plate under vertical vibration. Moreover, the dimer is able to respond to the plate’s vibration with various distinctive modes of self-propelled motion. This design is inspired by the earlier studies of a grain-filled bouncing ball on a vibrating or a static plate^19–21^, in which the coefficient of restitution and its resulting bouncing dynamics demonstrate rich dependence on the mass and size of grains inside the bouncing ball. In our work, we introduce an asymmetry to the dimer by differentiating the size of grains in the two balls, while keeping the total filling mass of grains as well as other mechanical properties in each ball identical. The key result is that without changing the grains or the design of the dimer, but instead varying the vibration parameters of the externally controlled plate, the dimer can be tuned into different modes of self-propulsion. This design specifically used in our experiments has an advantage compared with the existing active vibration driven self-propelled rod. The dynamics of the dimer here can be simplified into a set of a conservative force field, a combination of a symmetric and an asymmetric damping term that dissipate energy and break the symmetry of the dynamics.

**Figure 1:**
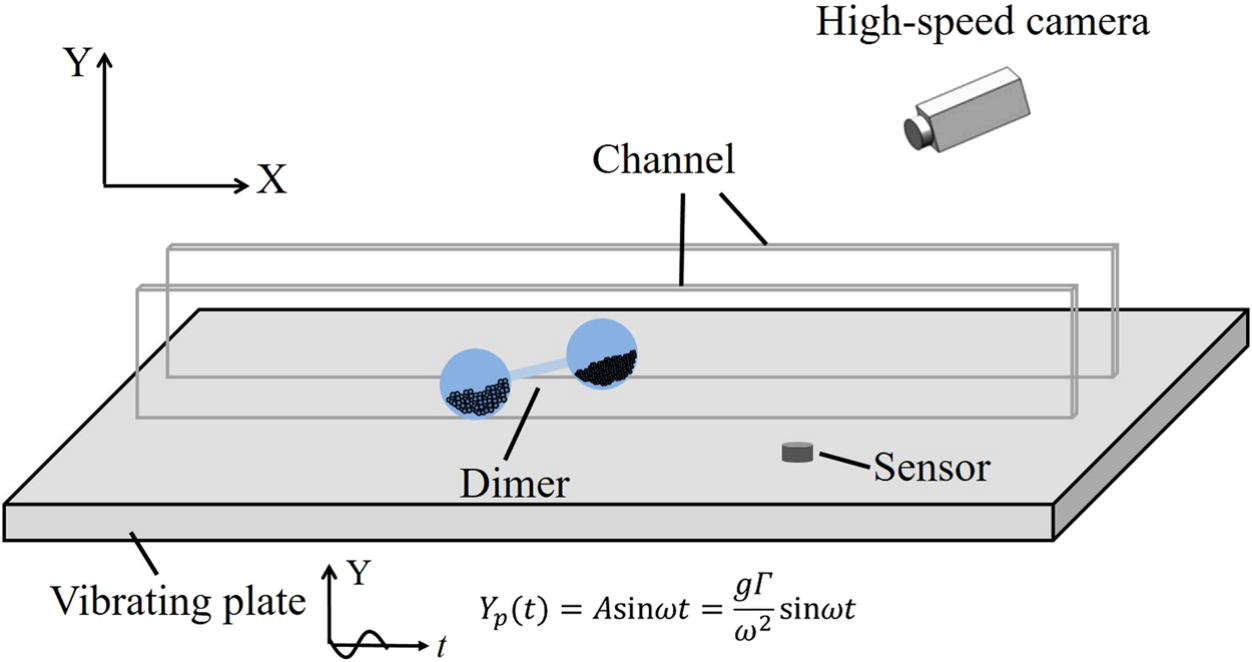
Side view of a grain-filled dimer in a quasi-one-dimensional, transparent channel attached to a vibrating plate. High-speed video is used to capture the motion of the active system. The horizontal direction *x* is along the channel, and the vertical direction *y* is perpendicular to the plane of vibrating plate. The dimer consists of two identical ping-pong balls rigidly connected by a hard rod; both balls are elastic and have the same friction constant with the plate; the balls are filled with the same mass of grains; the grain size of one ball, however, is different from that of the other.

In this manuscript, we mainly focus on two prominent types of self-propelled motion completely opposite directions by tuning the vertical vibration of the plate. First, we present the experimental results by illustrating the various modes of the active motion in a phase diagram parameterized by the frequency and the amplitude of the vibrating plate. To seek for physical insight, we conduct numerical simulation by simplifying the grain-filled ball into a ball connected to a damped harmonic oscillator, and find behavior consistent with the experimental observation. We show numerically that the damped dynamics results in a limit cycle regardless of the initial condition, and the shape of the limit cycle depends on both the amplitude and frequency of the vibrating plate. We also attempt to provide intuition into the cause of the biased motion. The experimental setup demonstrated in Fig. 1 is briefly described in the Method section. Readers are referred to Supplementary Information for more details of both experimental and numerical methods involved in this study.

## Results

The grain-filled dimer exhibits self-propelled, directed motion in a lateral quasi-one-dimensional channel under vertical vibration. It is found that, for the *same* dimer, the self-propulsion can reverse its direction as the vibration frequency, *f*, or the dimensionless acceleration, Γ of the vibrating plate is tuned. One mode is denoted as the toward-large-grain mode (TLG), where the net motion of the dimer is biased towards the ball filled with large grains (LG ball). Another mode is called the toward-small-grain (TSG) mode, where the net motion is towards the small-grain-filled ball (SG ball). The motions toward the LG ball and the SG ball are denoted by a positive-valued velocity and a negative-valued velocity, respectively.

We find that the self-propelled motion is not a consequence of initial conditions. The direction of motion is independent on the initial height, velocity, the initial orientation, angular velocity of the dimer, nor the initial phase of the external driving force (the controlled oscillatory motion of the vibrating plate). However, the direction of motion does depend on the size of the grains filled within each ball and the amplitude/frequency of the vibrating plate.

We conclude that the directed self-propulsion is neither an artifact due to the geometric or mass asymmetry of the balls nor the effect from any residual tilt of the plate. We note that the directed motion would vanish if the grain size is set the same in the two balls or if both balls are empty. In those situations, the dimer fluctuates in a stationary position. Consequently, we conclude that the symmetry breaking on the grain size is responsible for the directed active motion. This conclusion is confirmed by a numerical simulation of a simplified dimer exhibiting the same self-propelled motion as our experimental observation.

### Phase diagram

**Figure 2:**
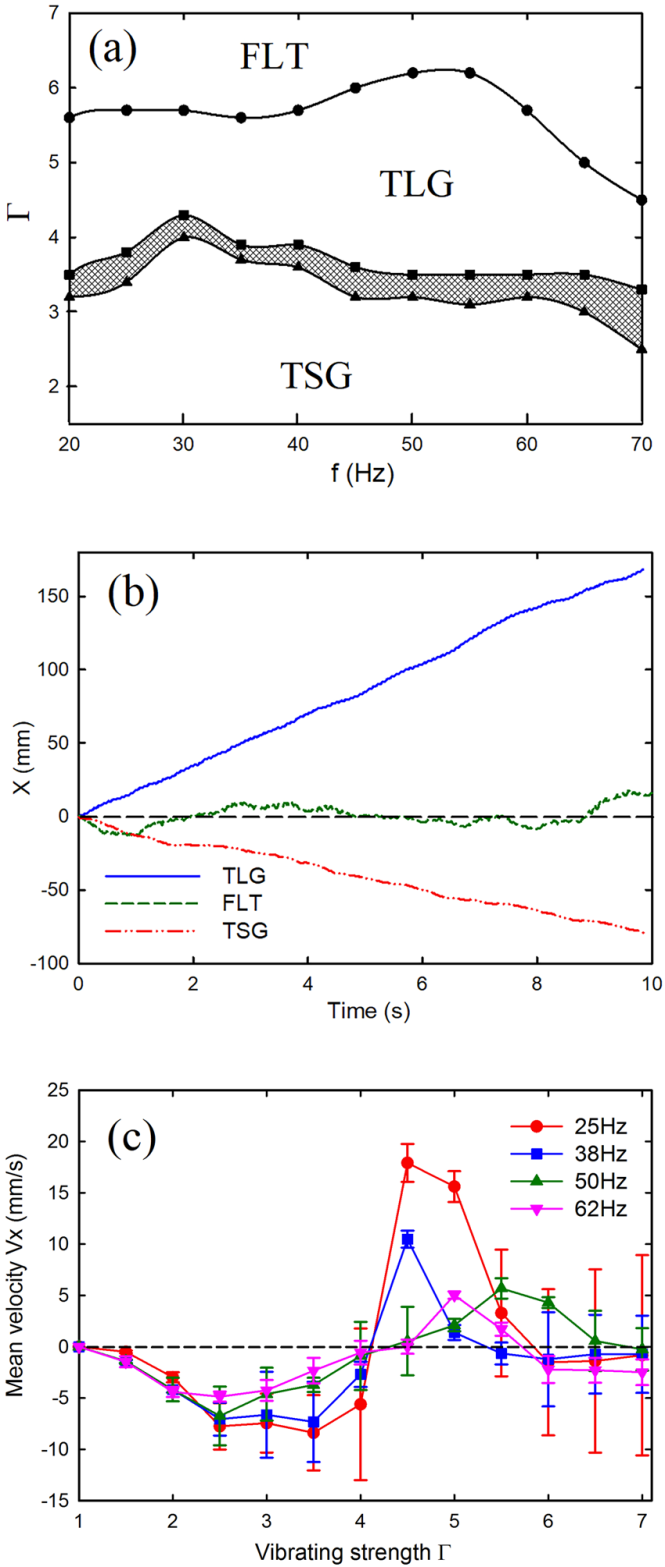
(**a**) A *f* − Γ phase diagram shows the regions of the observed motion of a partially filled dimer with, χ = 2.75, *m* = 12.91 g. Region TSG: self-propulsion towards the ball filled with small glass grains; TLG: self-propulsion towards the ball filled with large glass grains; the shaded area indicates a crossover in which either form of self-propulsion can happen. FLT: fluctuation, the dimer fluctuates randomly around its initial position. (**b**) Horizontal position of the geometric center of the dimer as a function of time for three modes in the phase diagram. TSG: *f* = 25 Hz, Γ = 2.5. TLG: *f* = 25 Hz, Γ = 4.5. FLT: *f* = 25 Hz, Γ = 6.5. (**c**) Mean horizontal velocity as a function of vibrating strength for different frequencies. All data points are averaged over 5 realizations.

A typical *f* *−* Γ phase diagram of the observed motion is shown in Fig. 2(a). In this phase diagram, the TSG mode can be found approximately within the range of Γ = 1.5–3.5 for all frequencies. On further increasing Γ to above 3.5, the TLG mode starts to emerge. There is a crossover regime, indicated by a shadowed area, between the TSG and TLG region, where the motion of the dimer is still directional, but the direction is stochastic for each trial. At higher frequencies, another regime appears, which we call fluctuation mode. In this case the dimer fluctuates randomly about its initial position within the time range of our observation. This implies that large Γ does not necessarily favor the occurrence of the TLG mode. With different filling mass and rod length, the TLG and TSG regions in the phase diagram differ, yet the phenomenon of directed migration is robust (see the supplementary materials). In this manuscript, we focus on the two directional self-propelled modes.

In Fig. 2(b), the horizontal displacements of the geometric center of a dimer over time for all three distinct modes are shown. In the TLG mode, the displacement increases with time since it moves toward the right-hand side which direction is defined as positive. The trajectory for TSG mode indicates that the dimer moves towards the opposite direction, and the mean speed is slower than that in TLG mode. For the fluctuation mode, the displacement fluctuates around the initial position, and the mean velocity approaches zero. Figure 2(c) shows the non-monotonic dependence of mean velocity on the vibrating strength for different frequencies.

It is known that the necessary conditions for the self-propulsion of active matter has the following characteristics: (1) The external environment supplies energy or the active matter itself stores energy. (2) Symmetry breaking must occur whereby active matter is capable of converting stored or ambient free energy into a systematically biased movement. In our system, the asymmetry lies in the sizes of the filling grains in the two balls, which causes an asymmetry in the energy dissipation within the two balls: for large grains, the inter-particle collision is mainly elastic, whereas for the small grains frictional force dominates the internal inter-particle collision^22–25^. It is therefore reasonable to speculate that different dissipation mechanisms inside the two balls result in dissimilar energy dissipation. The dissipative disparity gives rise to different vertical bouncing behaviors in the two balls with respect to the vibrating plate.

### Self-propulsion: TLG mode

**Figure 3:**
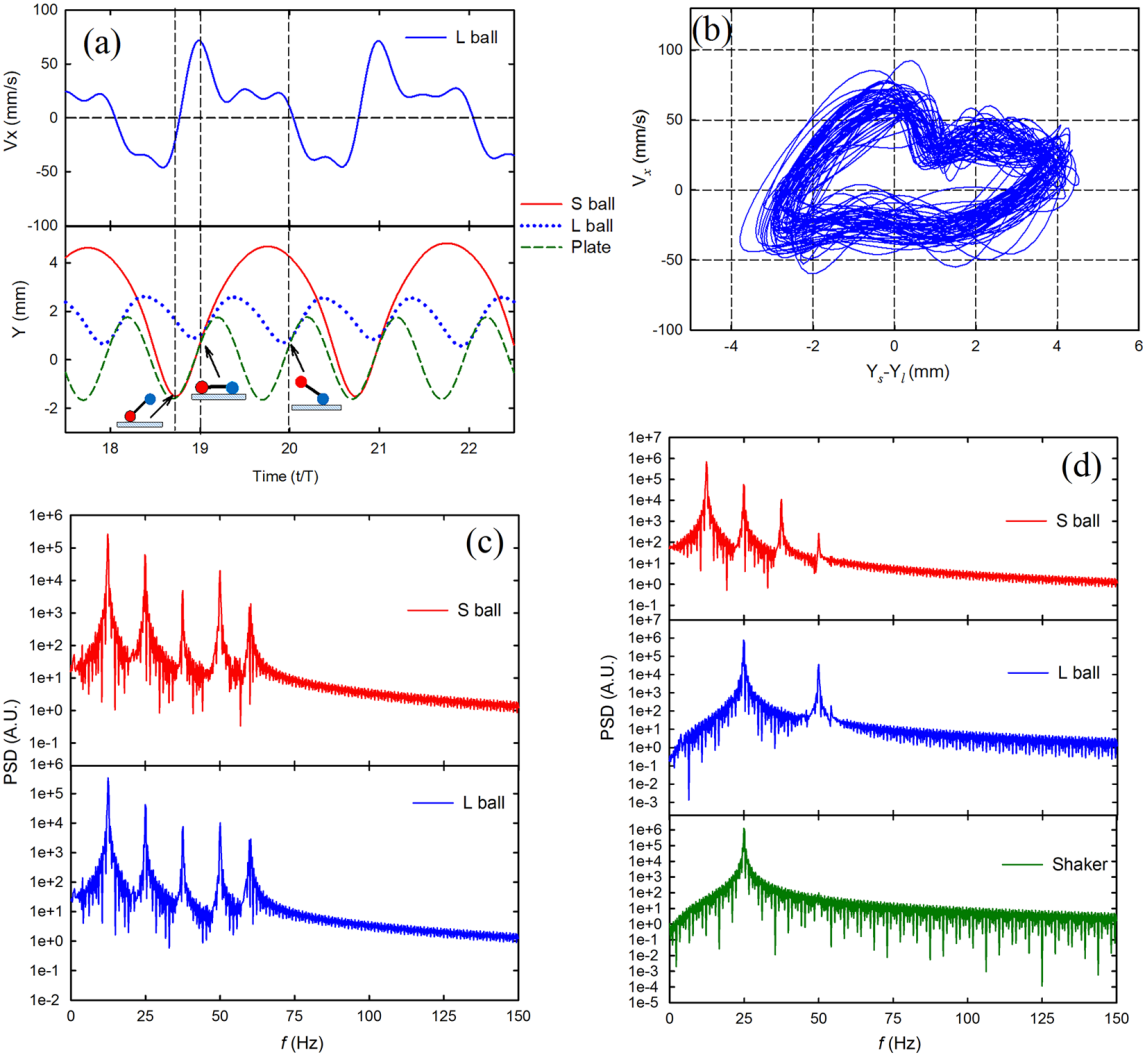
Statistical analysis on a typical TLG mode. χ = 2.75, *m* = 19.35 g, *f* = 25 Hz, Γ = 4.5. (**a**) Upper panel, instantaneous horizontal velocity of LG ball vs time. Due to the similarity, only the velocity of LG ball is shown. Lower panel, vertical position of both balls and the vibrating plate as a function of time. The cartoon drawing indicates the steric configuration of the dimer as the impact occurs. The red ball in the cartoon indicates the SG ball, and the blue ball is LG ball. (**b**) Horizontal velocity of LG ball vs the corresponding vertical distance difference between the SG and LG ball, forming a closed loop. (**c**) Power density spectrum of the horizontal velocity vs time. (**d**) Power density spectrum of the vertical velocity vs time.

The connection between the vertical bouncing and the horizontal drift velocity may shed more light on the self-propulsion. The vertical displacement in Fig. 3(a) displays a periodic bouncing behavior in a TLG mode where the LG ball (filled with large grains) collides every cycle with the plate whereas the SG ball (filled with small grains) collides with the plate only once every other vibration cycle. We note that the following behavior of the bouncing mode is general for the TLG mode. When a collision occurs in which the SG ball hits the plate while the LG ball is in the air, the SG ball maintains contact with the plate instead of bouncing immediately, as indicated by the leftmost cartoon in the lower panel of Fig. 3(a), which shows schematically the relative position of both balls. This is due to the loss of the kinetic energy dissipated by the filling grains. The horizontal velocity of LG ball rapidly varies from negative to positive after the impact, as shown in the upper panel of Fig. 3(a). During a typical collision of a ball with the vibrating plate, a large but short impulse of frictional force at the contact point causes the horizontal displacement after the collision^14,17^.

Subsequently, the LG ball collides with the plate as the SG ball is also on the plate, as indicated by the middle cartoon in the lower panel of Fig. 3(a). The corresponding horizontal velocity decreases after impact. Then the horizontal velocity remains approximately constant while the SG and LG ball detach from the plate. After a short span of time, the LG ball falls and impacts the plate but the SG ball is in flight, as shown in the rightmost cartoon in the lower panel of Fig. 3(a), and the direction of the horizontal velocity changes from positive to negative. Although in a given cycle the horizontal velocity induced by the collision with the resulting friction may be positive or negative, the mean horizontal velocity or net horizontal displacement is positive. Overall, each collision from the SG (LG) ball always contributes positive (negative) horizontal velocity to the motion of the dimer.

Figure 3(a) shows the features of collisional dynamics from several typical cycles. It is interesting and important to examine the global features during the entire self-propulsion. The relation of the relative height and corresponding horizontal velocity during a collision is shown to assess the global stability that indicates the repeatability of the bouncing dynamics in each bounce. In Fig. 3(b), a stable, closed orbit indicates a stable periodic orbit.

The velocity and vertical trajectories in the Fig. 3(a) are highly periodic, but it is difficult to directly observe all periodic ingredients which overlap or are distorted by stochastic noise. A power density spectrum (PDS) of the velocity is effective for analyzing the dimer motion. Figure 3(c) shows the PDS of the horizontal velocity, *V*_*x*_, of each ball in the TLG mode. Five sharp peaks at 12.5 Hz*n (*n* = 1, 2, 3, 4, 5) are clearly observed in the PDS, indicating five characteristic modes with stable periodicity in horizontal motion. The period doubling and halving in the PDS also indicate strong nonlinearity in the system. The height of a peak is proportional to the amplitude of the corresponding characteristic mode. The main peak, which has the largest height, shows at 12.5 Hz, at exactly half the driving frequency of the vibrating plate, confirms that the horizontal velocity undergoes a most important change every two vibrating cycles. The features of PDS between both balls are almost identical, implying that their horizontal motion shares a common mode since they are connected by a rigid rod.

Figure 3(d) shows the PDS of the vertical velocity, *V*_*y*_, of each ball and vibrating plate. There are four peaks showing for the SG ball and two peaks for the LG ball. The main peak in the SG ball is also 12.5 Hz, the same as the frequency of the predominant mode in horizontal motion. The same frequency implies that the collision from the SG ball contributes most to the horizontal motion. According to Fig. 3(c) and (d), it suggests the dimer converts the periodic, vertical momentum into a periodic, horizontal motion through the collision between the dimer and vibrating plate every two vibrating cycles.

### Self-propulsion: TSG mode

**Figure 4:**
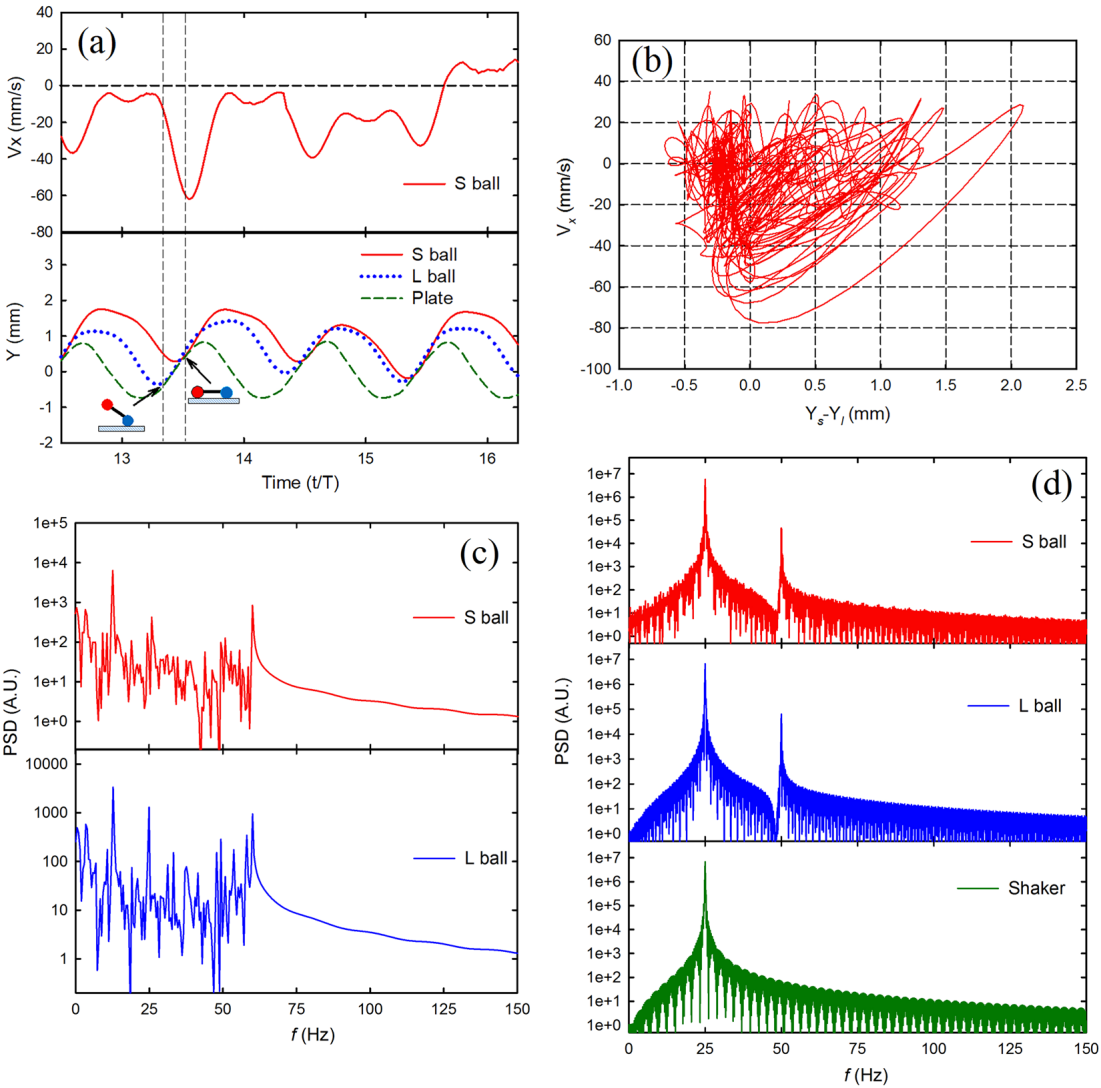
Statistical analysis on a typical TSG mode. χ = 2.75, *m* = 25.82 g, *f* = 25 Hz, Γ = 2.5. (**a**) Upper panel, instantaneous horizontal velocity of SG ball vs time. The lower panel, vertical position of both balls and the vibrating plate as a function of time. The cartoon drawing indicates the steric configuration of the dimer as it impacts the vibrating plate. (**b**) Horizontal velocity of SG ball vs the corresponding vertical distance difference between the SG and LG ball, showing irregularity. (**c**) Power density spectrum of the horizontal velocity vs time. (**d**) Power density spectrum of the vertical velocity vs time.

In the TSG mode, the vertical displacement also exhibits a nearly periodic bouncing mode in which both balls collide with the vibrating plate once every vibration cycle, see Fig. 4(a). One of the collisions occurs where the LG ball collides with the plate, and the SG ball is still in flight, as shown by the leftmost cartoon in the lower panel of Fig. 4(a). After the impact, the LG ball moves vertically together with the plate, and the horizontal velocity drops. Shortly after the collision, the SG ball impacts the plate as the LG ball still rests on the plate, as indicated by the rightmost cartoon in the lower panel of Fig. 4(a). During this collision, the horizontal velocity increases. The horizontal velocity during the collision is mainly determined by a series of parameters, such as the collisional location at the plate, the relative height of both balls and the horizontal velocity just before the collision. Unlike the TLG mode, the amplitude of the horizontal velocity in the TSG mode has wide variation at each collision, which implies that these parameters differ greatly during each collision.

Compared with Fig. 3(b), the horizontal velocity of the TSG mode from each collision varies, as shown in Fig. 4(b). Remarkably, the collisional order persists for most vibration cycles (namely both balls are in air, LG ball collides but SG ball is in flight, SG ball collides as LG ball is on the plate, then the procedure repeats). However, an unusual situation where the SG ball collides with the plate but the LG ball is in flight occasionally occurs. Although such a change in the collisional order returns to its “normal” state soon, the reversal in the order of the collisions causes the apparent aperiodic behavior in the Fig. 4(b).

Although the horizontal velocity *V*_*x*_ in Fig. 4(a) seems to lack periodicity, Fig. 4(c) exhibits the PDS of *V*_*x*_ for each ball in the TSG mode. Several peaks can be found, but are smaller than those in Fig. 3(c). The frequency of the main peak is located at 12.5 Hz. The PDS in the vertical velocity is similar with the TLG mode, except that the frequency in the SG ball is 25 Hz, as shown in Fig. 4(d). It is in accordance with the fact that the SG ball always collides with the plate once per vibration cycle.

The phase diagram in Fig. 2(a) can qualitatively support the results in Figs 3 and 4. The TSG mode mainly occurs at the region of low Γ, and the TLG mode occupies the region of high Γ. The energy *E* transferred from the shaker to the dimer at each vibration cycle is proportional to Γ, namely $E \sim {A}^{2} \sim {{\rm{\Gamma }}}^{2}/{\omega }^{4}$. At a given frequency, large Γ means that enough energy can excite the SG and LG ball to bounce higher, and make it possible to collide with vibrating plate once per two consecutive cycles. However, excessive Γ can also lead to loss of the periodicity and unstable bouncing behavior in the vertical direction. Similarly, in the region within high *f* or weak Γ, both balls, due to insufficient energy imparted from the shaker, fail to bounce high and thus the TSG mode almost always occurs in that region.

## Theoretical Model and Numerical Simulations

To gain some physical insight behind the biased active motion, namely the directed self-propulsion observed during the experiment, we construct a simplified model of the grain-filled-ball dimer, referring to previous work on similar theoretical models that allow to connect the geometrical and dynamical properties of an asymmetric object to its propulsion^26–28^, and demonstrate similar tunable biased active motion through numerical simulation. The simulation reveals biased active motion with asymmetric damping.

**Figure 5:**
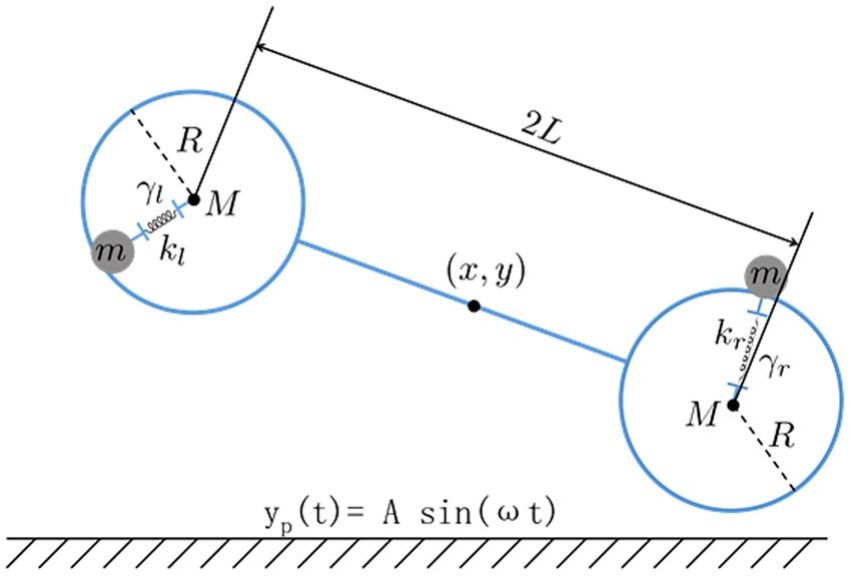
Illustration of the model of the grain-filled dimer. The dimer consists of 3 rigid bodies: A rigid dimer (blue dumbbell-shaped object) and two identical point particles of mass *m* (gray solid circles) connected to the dimer by two dashpots. The dimer is made of two identical balls (blue circles) of mass *M* and they are glued to the two ends of a massless and rigid rod. The point particle interacts with the dimer through a dashpot connected to the center of each ball. The asymmetry of the composite system only exists within the friction coefficient of the two dashpots.

Consider two identical elastic balls (of the same mass *M*) that are connected by a rigid massless rod as a dumbbell-shaped rigid body. The mass center of the rigid dumbbell is located at the center of the rod. Rather than modelling the filled grains in each ball, we simplify the grains into two damped harmonic oscillators (of the *same* mass *m*, *same* spring constant *k* but *different* damping friction coefficient *γ*_*l*_ and *γ*_*r*_) connected to the two geometric centers of the two elastic balls (See Fig. 5).

The (impulsive) elastic supportive force between the lowest point of the dumbbell and the surface of the vibrating plate is modeled as

1 $${Z}_{l,r}=S\cdot n\cdot {\Theta }({y}_{p}+R-{y}_{l,r})\cdot {(1+{y}_{p}+R-{y}_{l,r})}^{n-1}$$

where *Θ*(·) is the Heaviside step function, and *S* is a constant, and *n* is an even number. One can verify that the conservative potential corresponding to this support force represents a hard wall interaction in the limit of *n* → *∞*. Also note that the height of the vibrating plate *y*_*p*_ is oscillating with time. The friction force at the contact point between the ball and the plate can thus be written as

2 $${F}_{l,r}^{friction}=-sign({v}_{slidel,r})\cdot \mu \cdot {Z}_{l,r}$$

where ${v}_{{slide\; l},r}$ is the relative horizontal velocity of the contact point between the ball and the plate surface, and $\mu$ is the coefficient of friction between the ball and the plate. Note that the damped harmonic oscillator connecting each point mass *m* with each ball generates the following force at the center of the elastic balls:

3 $${F}_{l,r}^{friction}=-k(\vec{{r}_{l,r}^{os}}-\vec{{r}_{l,r}^{ball}})-{\gamma }_{l,r}\cdot (\vec{{v}_{l,r}^{os}}-\vec{{v}_{l,r}^{ball}})$$

where *k* is the spring constant, ${\gamma }_{l,r}$ is the friction coefficient for damping, ${\overrightarrow{r}}_{l,r}^{os}$ is the location of both oscillators, ${\overrightarrow{r}}_{l,r}^{ball}$ is the location of the centers of both balls, ${\overrightarrow{v}}_{l,r}^{os}$ is the velocity of both oscillators, and ${\overrightarrow{v}}_{l,r}^{ball}$ is the velocity of the centers of both balls. The only difference between the left ball and the right ball is the difference in their internal damping *γ*_*l*,*r*_. The point mass *m* does not interact with the boundary of the elastic ball or the vibrating plate.

With this minimal model of the asymmetric dimer, we numerically integrate the equations of the motion

4 $$\frac{dx}{dt}={v}_{x}$$

5 $$\frac{dy}{dt}={v}_{y}$$

6 $$2M\frac{d{v}_{x}}{dt}={F}_{xol}+{F}_{xor}+{F}_{l}^{friction}+{F}_{r}^{friction}$$

7 $$2M\frac{d{v}_{y}}{dt}={F}_{yol}+{F}_{yor}+{Z}_{l}+{Z}_{r}+2Mg$$

Where ${F}_{xol}$, ${F}_{xor}$, ${F}_{yol}$, and ${F}_{yor}$ are the force ${F}_{l,r}^{{interier}}$ projected to the *x* (or *y*) axis. In addition to the translational degrees of freedom, the rigid part of the dimer also has a rotational degree of freedom. The corresponding equations of motion can be written as

8 $$\frac{d\theta }{dt}=\omega $$

9 $$\frac{d\omega }{dt}=\frac{1}{2M{L}^{2}}(LO+RO+LT+RT)$$

10 $$LO=-L\,\cos \,\theta ({F}_{yol}-Mg)+L\,\sin \,\theta {F}_{xol}$$

11 $$RO=L\,\cos \,\theta ({F}_{yor}-Mg)-L\,\sin \,\theta {F}_{xor}$$

12 $$LT=-L\,\cos \,\theta {Z}_{l}+(L\,\sin \,\theta +R){F}_{l}^{friction}$$

13 $$RT=L\,\cos \,\theta {Z}_{r}-(L\,\sin \,\theta -R){F}_{r}^{friction}$$

The two oscillators connected by the dashpot to the left and the right balls are dictated by the equations of motion independent from the rigid body. The oscillators’ dynamics are determined by their gravitational force, the restoring force from the spring and the friction damping force, which are not written explicitly here. By integrating the equations of motion for the rigid dimer and the two point masses numerically, we demonstrate similar behavior to what was found in the experiment. The set of parameters we use in our simulations are:

Diameter of the ping-pong ball is *D* = 40 mm, mass of each ping-pong ball is *M* = 3.6 g, rod length 2*L* = 40 mm, mass of each harmonic oscillator *m* = 12.9 g. The above four parameters are chosen to match the design from the experiment. The parameters of the two dashpots are chosen as *k*_*l*_ = 1 *N/m*, *γ*_*r*_ = 10 *N* · *s/m*, *k*_*r*_ = 1 *N/m*, *γ*_*r*_ = 2 *N* · *s/m*. These four parameters are determined by fitting simulation results of a single ping-pong ball with a dashpot to the experimental observation of a single grain-filled ball on a vibration plate. Other parameters used in the simulations are *S* = 50 *N/m*; *n* = 4; *μ* = 0.2. For accuracy, we use a very small integration time step Δt = 50 ns.

**Figure 6:**
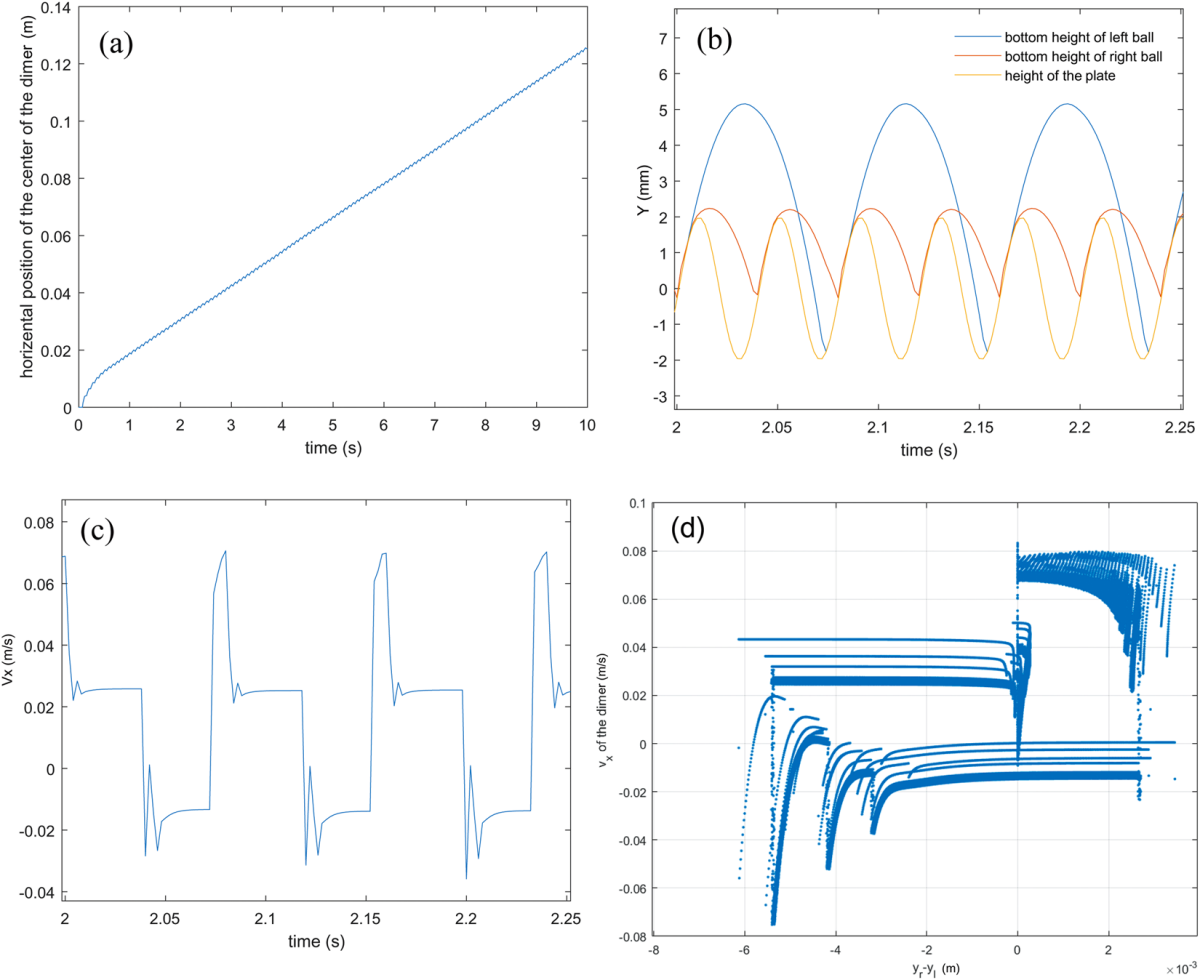
(**a**) Simulation result from the simplified model in the motion toward the large-grain-filled ball (TLG mode). *f* = 25 Hz, Γ = 2.5. (**b**) The blue, red and yellow lines represent the vertical position of SG filled ball, LG filled ball and the vibrating plate as a function of time. The vertical bouncing mode from the simulation is very similar with the experimental observation. (**c**) Instantaneous horizontal velocity of LG ball vs time. (**d**) Horizontal velocity of LG ball vs the corresponding vertical distance difference between the SG and LG ball.

**Figure 7:**
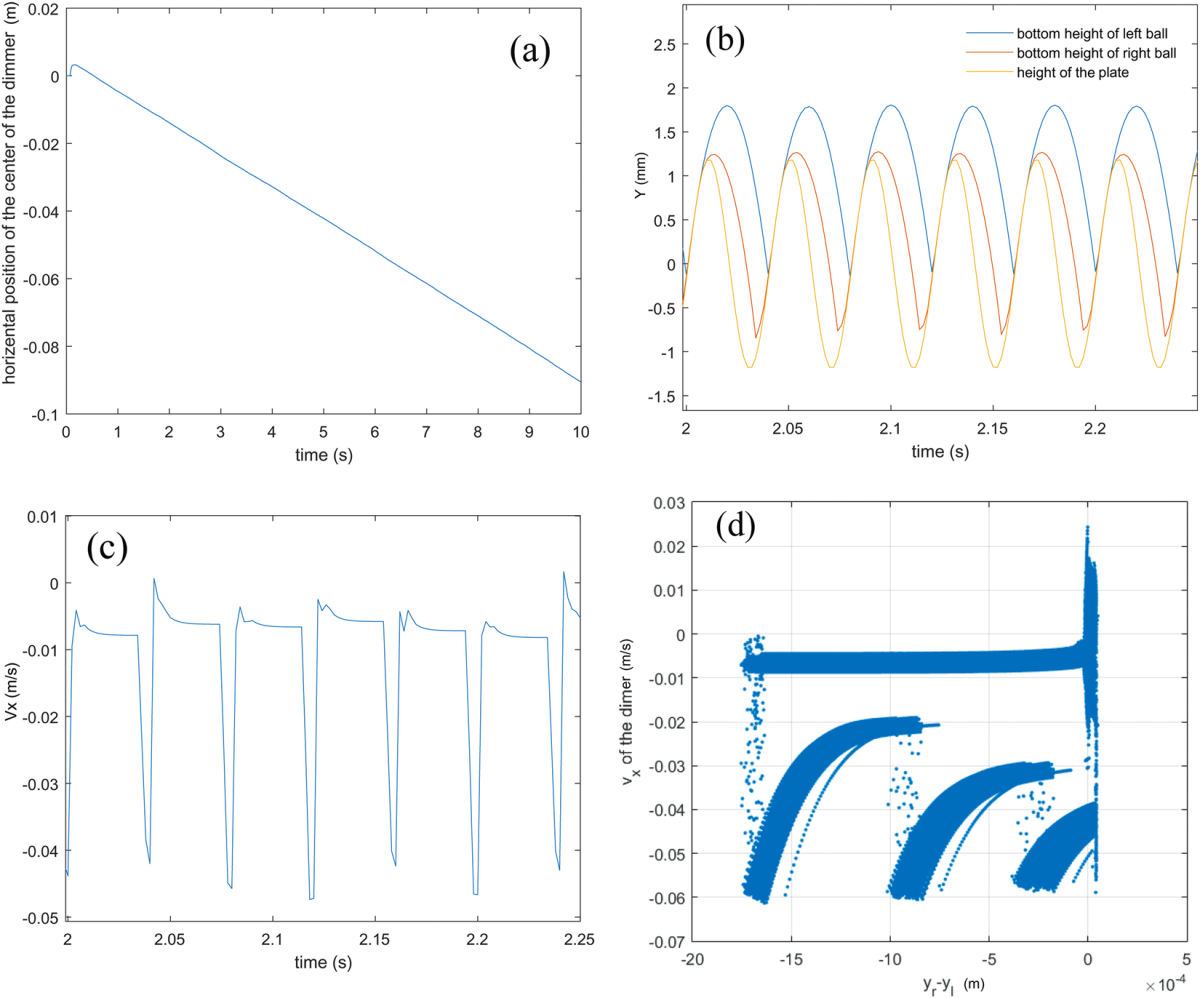
(**a**) Simulation from the simplified model in the motion toward the small-grain-filled ball (TSG mode). The parameters in Fig. 7 are exactly the same as those in Fig. 6 except for Γ = 2.5. (**b**) The blue, red and yellow lines represent the vertical position of SG filled ball, LG filled ball and the vibrating plate as a function of time, respectively. (**c**) Instantaneous horizontal velocity of SG ball vs time. (**d**) Horizontal velocity of SG ball vs the corresponding vertical distance difference between the SG and LG ball.

We compare the behaviors of the simplified model with the experiment, and find that they both have tunable biased active motion. See Figs 3, 4, 6 and 7 for the comparison of the moving modes of the numerical simulation and the experiment. The basic features from the simulation agree well with the experiment. The only difference in the simulation and the experiment is the vertical distance difference dependence of the horizontal velocity of in the TSG mode (Figs 7(d) and 4(b)). The simulation does not show an irregular form in Fig. 7(d) but demonstrate a periodic orbit. It presumably results from the oversimplification of the filling grains that filters out other sources of noise from its dynamics. Despite this, the agreement indicates that although the model is simple and coarse, it captures the essence of the underlying physics in the self-propulsion.

## Discussion

**Figure 8:**
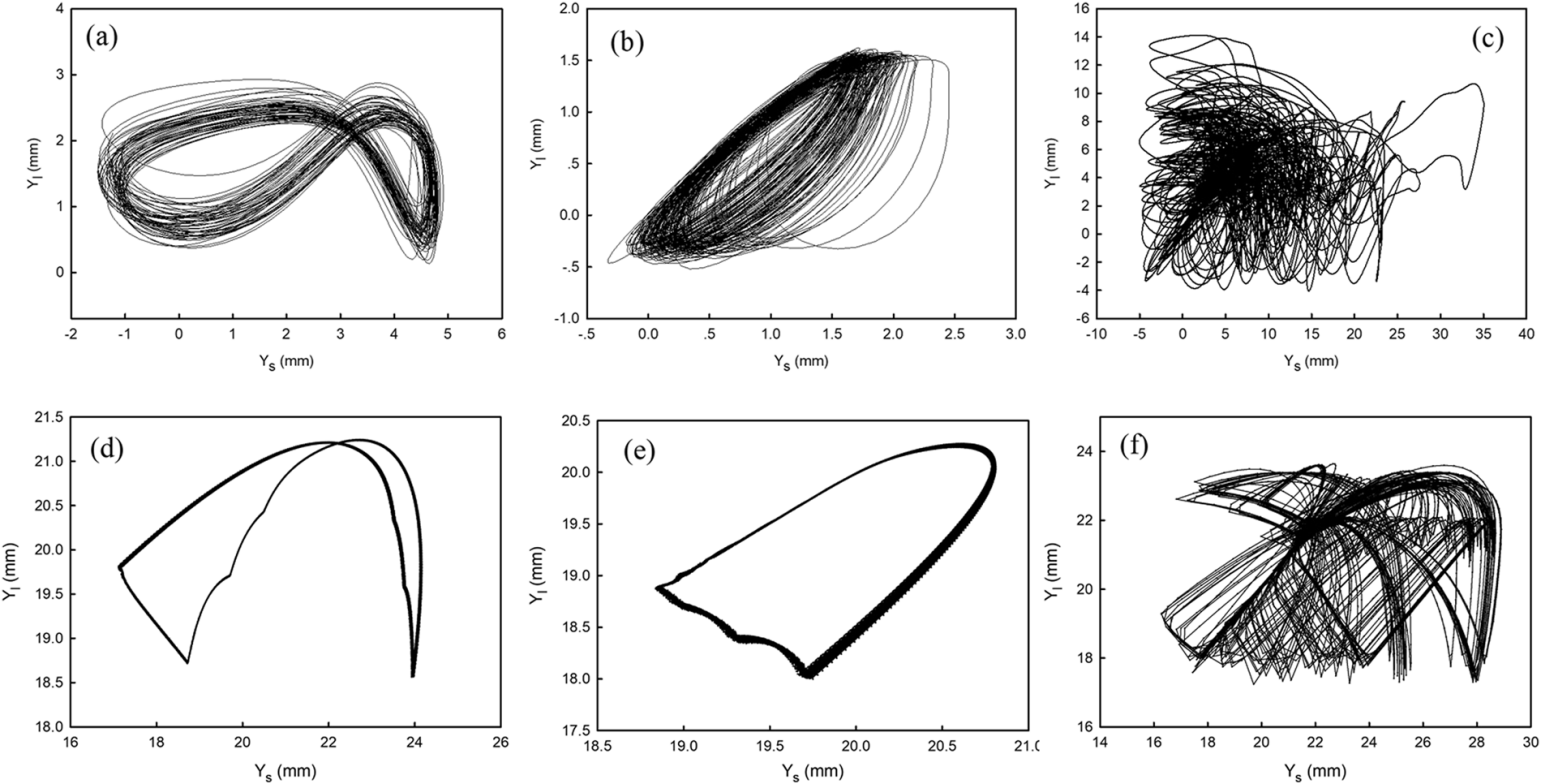
Comparison of experiments and simulations of the vertical position of each ball, Y_*s*_ (small-grain-filled ball) vs Y_*l*_ (large-grain-filled ball) (**a**–**c**) Experimental results of TLG mode, TSG mode, and fluctuation mode, respectively. (**d**–**f**) Simulations of TLG mode, TSG mode, and fluctuation mode, respectively.

Through numerical simulation of the TLG and TSG mode, we confirm that each mode corresponds to a limit cycle attractor. Regardless of the initial condition, the TSG mode converges into a periodic orbit with two collisions per cycle whereas the TLG mode converges into a figure-eight periodic orbit with three collisions per period. These periodic orbits are shown in Fig. 8(a,b,d and e).

Beyond the directed self-propulsion regions, at least one of the balls loses periodicity in the vertical bounce and shows irregular bouncing behavior in the FLT regime. In this case, the numerical simulation does not converge into a periodic orbit, (See Fig. 8(c) and (f)). As described above, the dimer in the state fluctuates randomly, and the definite directionality disappears. More precisely, as long as either ball loses periodicity in its vertical motion, the dimer starts to fluctuate. See the supplementary materials. For the directed self-propulsion, periodic bounce for both balls is necessary; for the fluctuation mode, at least one ball must lose its periodicity in its vertical motion.

These analyses summarize the statistical features for two types of self-propulsion, and reveal that the vertical bouncing behavior of both balls has a crucial influence on the directed self-propulsion. It notes that although the conclusion is summarized from two typical TLG and TSG modes, the rule is general for all modes in our experiments. The supplementary materials present data from the TLG and TSG modes with different rod lengths and filling masses.

In summary, we have built an “active” dimer partially filled with granular particles with the same material properties except for their grain size. We find that such a grain-filled dimer exhibits self-propelled, directed motion under vertical vibration. By tuning vibration conditions, two types of directed self-propulsion with opposite direction from the *same* dimer are observed. We further investigate relevant dynamical properties of the self-propulsion in details.

We also present a simplified model and reproduce the tunable self-propulsion by numerical simulation. The simulation results agree well with those in the experiment. The simulation discovers that the dissipative disparity in the balls is responsible for the directed motion. We speculate that asymmetry on the particle size could result in dissimilar energy dissipation on each ball due to different energy dissipation mechanisms during vibration. When the external excitation is appropriate, the synchronous coupling between the driven vibration and vertical bounce of each ball can generate a stable SG (LG) ball-vibrating plate collision. Through the collision between the dimer and vibrating plate, the dimer converts the periodic momentum in vertical direction into a periodic, horizontal momentum. For the fabrication of shaken active matters, periodic bouncing in the vertical direction for both balls seems to be a favorable condition.

## Methods

The grain-filled dimer used in our experiment consists of two identical ping-pong balls connected by a light, rigid rod. The connecting rod firmly fixed between two balls is not retractable. The aspect ratio of a dimer is defined as $\chi =\frac{2D+L}{D}$, where *L* is the length of the rod, and *D* the outer diameter of the ping-pong ball. The mass of each ping-pong ball is *m* = 36.1 ± 0.03 grams, the outer and inner diameter are *D* = 39.5 ± 0.05 mm and *d* = 38.0 ± 0.05 mm, respectively. The coefficient of restitution of the hollow ball is *e* = 0.75 ± 0.02 when it drops and collides with the plate. During the experiment, the two balls are partially filled by glass beads with the same filling mass but with different diameter: *d*_1_ = 3 ± 0.1 mm, and *d*_2_ = 0.3 ± 0.03 mm.

The grain-filled dimer is placed between two acrylic walls that are rigidly attached to a metal plate and spaced by 41 mm to form a quasi-one-dimensional channel, as shown in Fig. 1. The plate is driven by a shaker with vertically sinusoidal vibration, *Y*(*t*) = *A* sin *ωt*. Two control parameters, the frequency *f* and the dimensionless acceleration Γ = *Aω* ^2^/g., generally characterize the vibration, where *ω* is the angular frequency, *A* the vibration amplitude, and *g* the acceleration due to gravity. A high-speed camcorder (Phantom V7.3) is used to record the bouncing dimer. Imaging algorithms can extract the trajectories of both ping-pong balls separately.

## Electronic supplementary material

S1

S2

S3

Supplementary materials
