# Supplementary material for: Self-propulsion of a grain-filled dimer in a vertically vibrated channel
Source: Sci Rep. 2017 Oct 27;7:14193. doi: 10.1038/s41598-017-14299-8 (PMC5660184; doi:10.1038/s41598-017-14299-8)
Supplement: Supplementary file 4 — Supplementary materials [file 41598_2017_14299_MOESM4_ESM.pdf]

Supplemental Material for Self-propulsion of a grain-filled dimer in a vertically vibrated channel

C. Xu,[1] N. Zheng,[1,2*] L-P Wang,[1] L-S Li,[3†] Q-F Shi and Zhiyue Lu[4**]

[1] *School of Physics, Beijing Institute of Technology, Beijing 100081, China*

[2] *Key Laboratory of Cluster Science of Ministry of Education, Beijing 100081, China*

[3] *Science and Technology on Electromagnetic Scattering Laboratory, Beijing 100854, China*

[4] *James Franck Institute, University of Chicago, Chicago 60637, USA*

I. Ratchet-like behavior of horizontal displacement in TLB mode

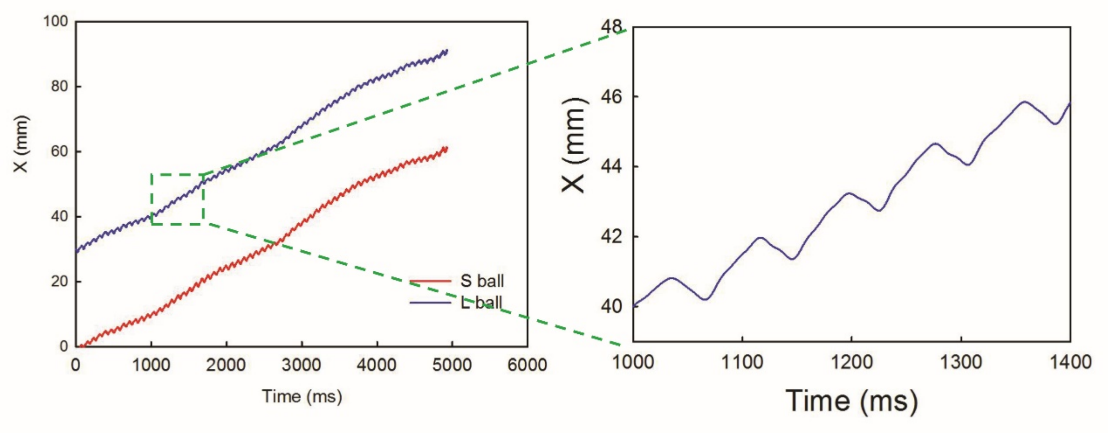

Fig.S1 Horizontal displacement in TLB mode increases with time, showing a ratchet-like characteristic at a short time scale. The enlarged part on the right exhibits the details.

In contrast to TSB mode, the regular ratchet feature in the displacement of TLB clearly appears. This implies that the direction of the horizontal velocity of the dimer alternatively changes. The regularity indicates that the motions of dimer and vibrating plate synchronize such that the collision between them is highly repeatable.

II. $f-\Gamma$ Phase diagram

* Ningzheng@bit.edu.cn
†liliangsheng@gmail.com
**zhiyuelu@gmail.com

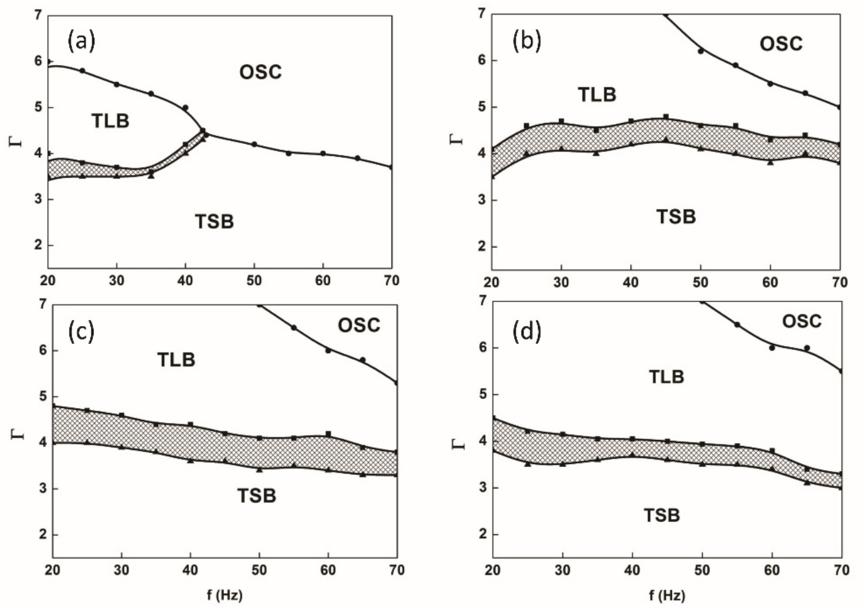

Fig.S2 $f-\Gamma$ phase diagrams to represent self-propelled motion are shown for various dimers. (a) $\chi = 2.25$, $m = 12.91g$ ($\zeta = 1/4$); (b) $\chi = 3.25$, $m = 12.91g$ ($\zeta = 1/4$); (c) $\chi = 3.75$, $m = 19.35g$ ($\zeta = 3/8$); (d) $\chi = 3.75$, $m = 12.91g$ ($\zeta = 1/4$).

We use different filling masses, aspect ratios of dimers to illustrate that the self-propulsion (TLB and TSB) is not a coincidence for some peculiar dimer, which always occurs for all dimers in our experiment. It is also noteworthy that the TLB and TSB regions in the phase diagram may vary for different dimers.

III. Instantaneous horizontal velocity of L (S) ball and corresponding vertical bouncing mode

(A) TLB mode

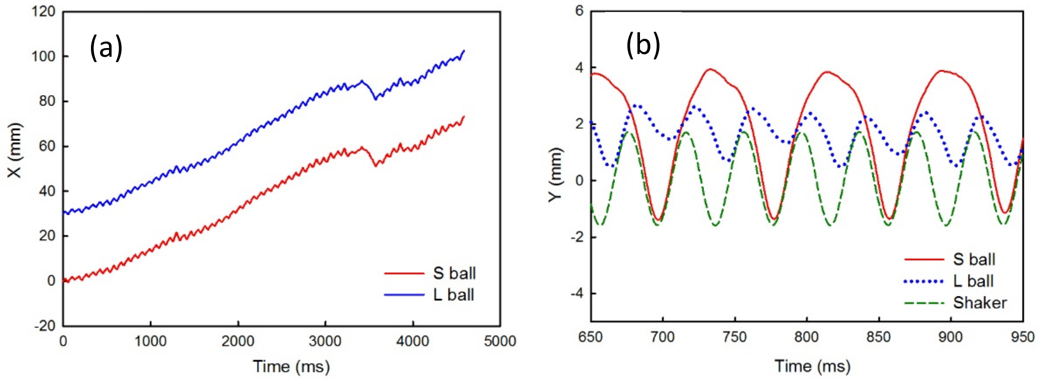

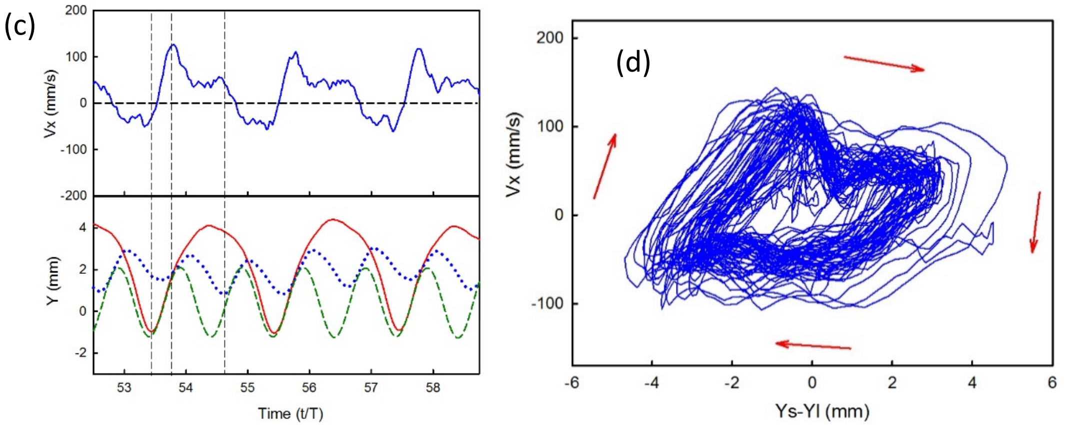

Fig.S3 TLB mode for a dimer with $\chi = 2.25$, $m = 12.91g$ ($\zeta = 1/4$), f=25 Hz, $\Gamma = 4.5$. (a) Horizontal position of the spherical centers of both ping-pong balls as a function of time. (b) Vertical position of the spherical centers of the both ping-pong balls and the vibrating plate as a function of time. (c) Upper panel, instantaneous horizontal velocity of L ball vs time in a typical TLB mode. Lower panel, vertical position of the both ping-pong balls and the vibrating plate as a function of time. Red solid curve: S ball; blue dotted curve: L ball; green dashed curve: shaker. The straight dashed line indicates the impact location. (d) Curves of horizontal velocity of L ball vs the vertical distance difference between the S and L ball for a complete measurement in (c), forming a closed loop.

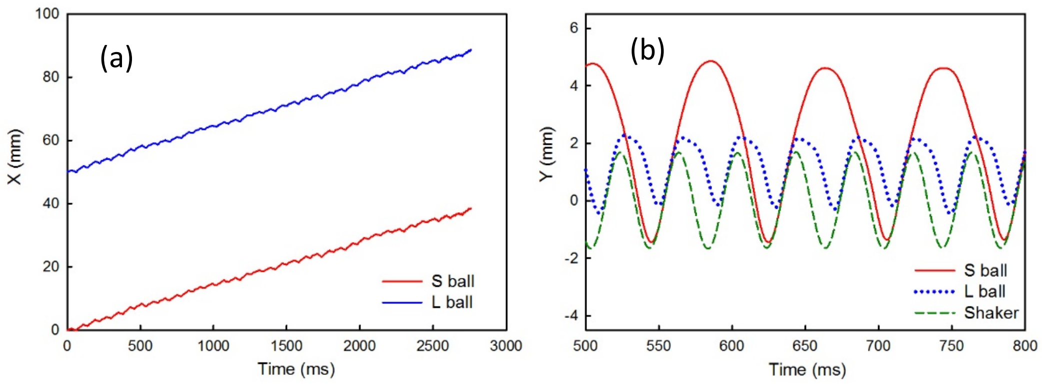

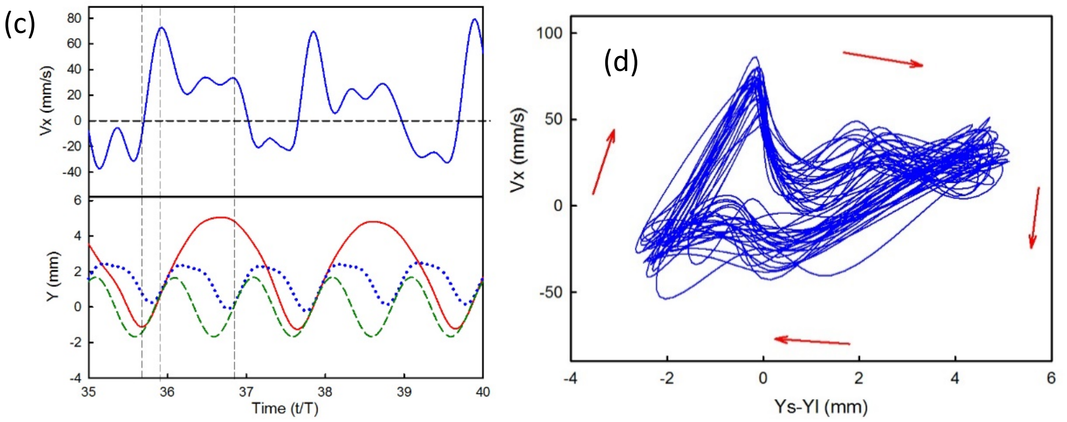

Fig.S4 TLB mode for a dimer with $\chi = 3.25$, $m = 12.91g$ ($\zeta = 1/4$), f=25 Hz, $\Gamma = 4.5$. (a) Horizontal position of the spherical centers of both ping-pong balls as a function of time. (b) Vertical position of the spherical centers of the both ping-pong balls and the vibrating plate as a function of time. (c) Upper panel, instantaneous horizontal velocity of L ball vs time in a typical TLB mode. Lower panel, vertical position of the both ping-pong balls and the vibrating plate as a function of time. Red solid curve: S ball; blue dotted curve: L ball; green dashed curve: shaker. The straight dashed line indicates the impact location. (d) Curves of horizontal velocity of L ball vs the vertical distance difference between the S and L ball for a complete measurement in (c), forming a closed loop.

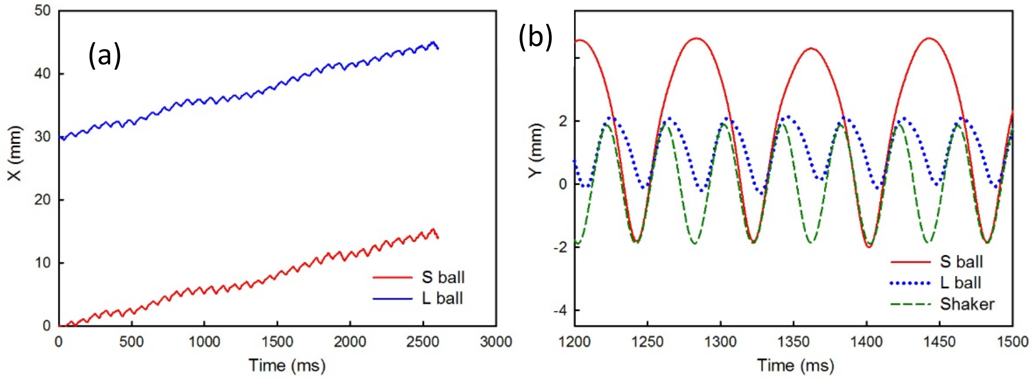

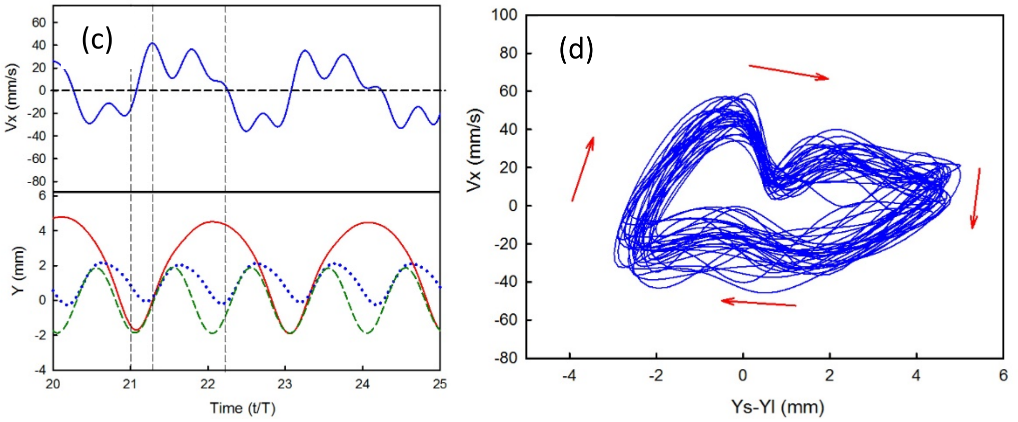

Fig.S5 TLB mode for a dimer with $\chi = 3.75$, $m = 19.35g$ ($\zeta = 3/8$), f=25 Hz, $\Gamma = 5$. (a) Horizontal position of the spherical centers of both ping-pong balls as a function of time. (b) Vertical position of the spherical centers of the both ping-pong balls and the vibrating plate as a function of time. (c) Upper panel, instantaneous horizontal velocity of L ball vs time in a typical TLB mode. Lower panel, vertical position of the both ping-pong balls and the vibrating plate as a function of time. Red solid curve: S ball; blue dotted curve: L ball; green dashed curve: shaker. The straight dashed line indicates the impact location. (d) Curves of horizontal velocity of L ball vs the vertical distance difference between the S and L ball for a complete measurement in (c), forming a closed loop.

Fig.S3-5 shows the kinetic features in TLB mode for different dimers and vibrating conditions. Although there are some differences in details such as the horizontal velocity after collision in Fig. S3-5(c), and curve shapes in Fig. S3-5(d), all TLB modes share common kinetic features shown in these figures. The arbitrary choice of dimers together with the common features in Fig. S3-5 suggest the features are robust and the same underlying mechanism for all dimers dominate the TLB mode.

(B) TSB mode

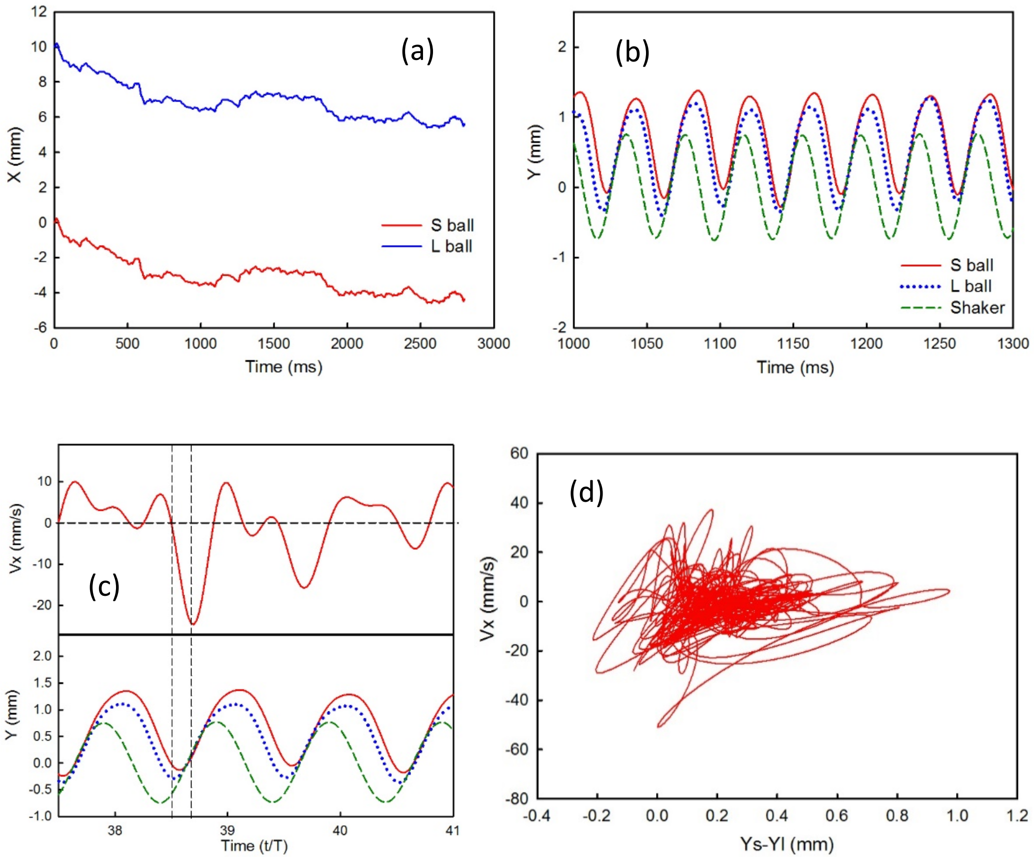

Fig.S6 TSB mode for a dimer with $\chi = 2.25$, $m = 12.91g$ ($\zeta = 1/4$), f=25 Hz, $\Gamma = 2$. (a) Horizontal position of the spherical centers of both ping-pong balls as a function of time. (b) Vertical position of the spherical centers of the both ping-pong balls and the vibrating plate as a function of time. (c) Upper panel, instantaneous horizontal velocity of S ball vs time in a typical TSB mode. Lower panel, vertical position of the both ping-pong balls and the vibrating plate as a function of time. Red solid curve: S ball; blue dotted curve: L ball; green dashed curve: shaker. The straight dashed line indicates the impact location. (d) Curves of horizontal velocity of S ball vs the vertical distance difference between the S and L ball for a complete measurement in (c), showing in an erratic manner.

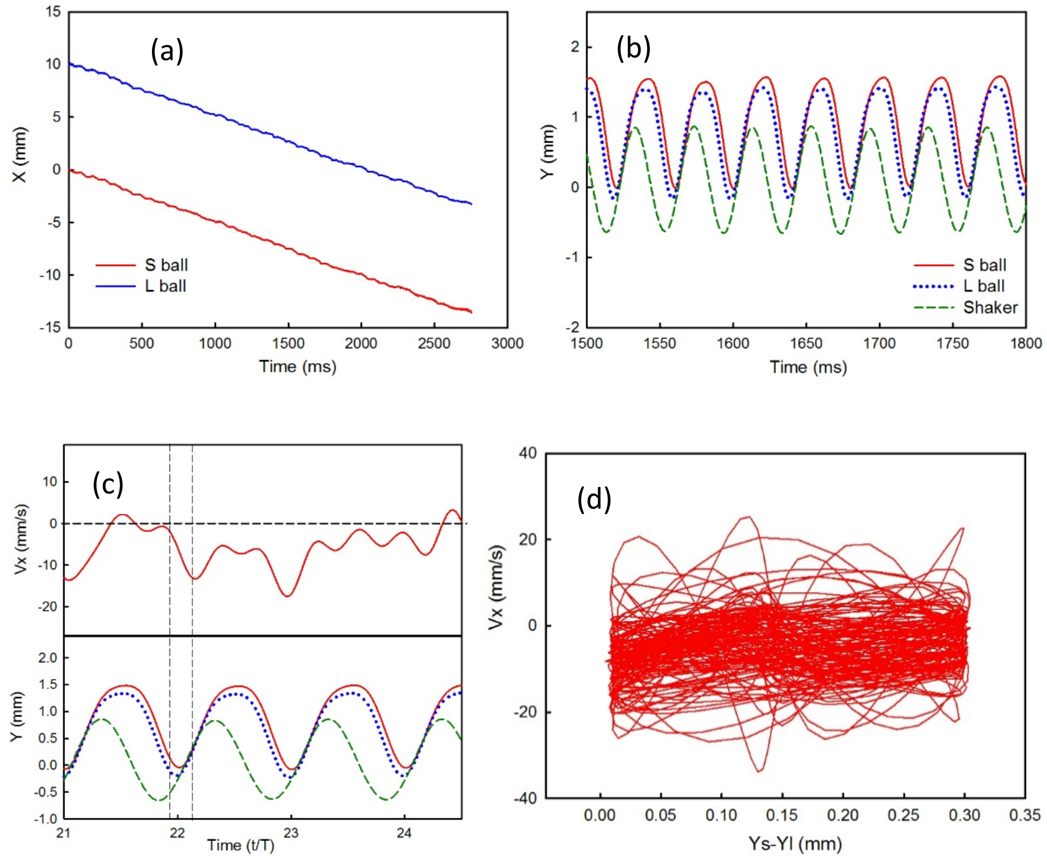

Fig.S7 TSB mode for a dimer with $\chi = 3.25$, $m = 12.91g$ ($\zeta = 1/4$), f=25 Hz, $\Gamma = 2$. (a) Horizontal position of the spherical centers of both ping-pong balls as a function of time. (b) Vertical position of the spherical centers of the both ping-pong balls and the vibrating plate as a function of time. (c) Upper panel, instantaneous horizontal velocity of S ball vs time in a typical TSB mode. Lower panel, vertical position of the both ping-pong balls and the vibrating plate as a function of time. Red solid curve: S ball; blue dotted curve: L ball; green dashed curve: shaker. The straight dashed line indicates the impact location. (d) Curves of horizontal velocity of S ball vs the vertical distance difference between the S and L ball for a complete measurement in (c), showing in an erratic manner.

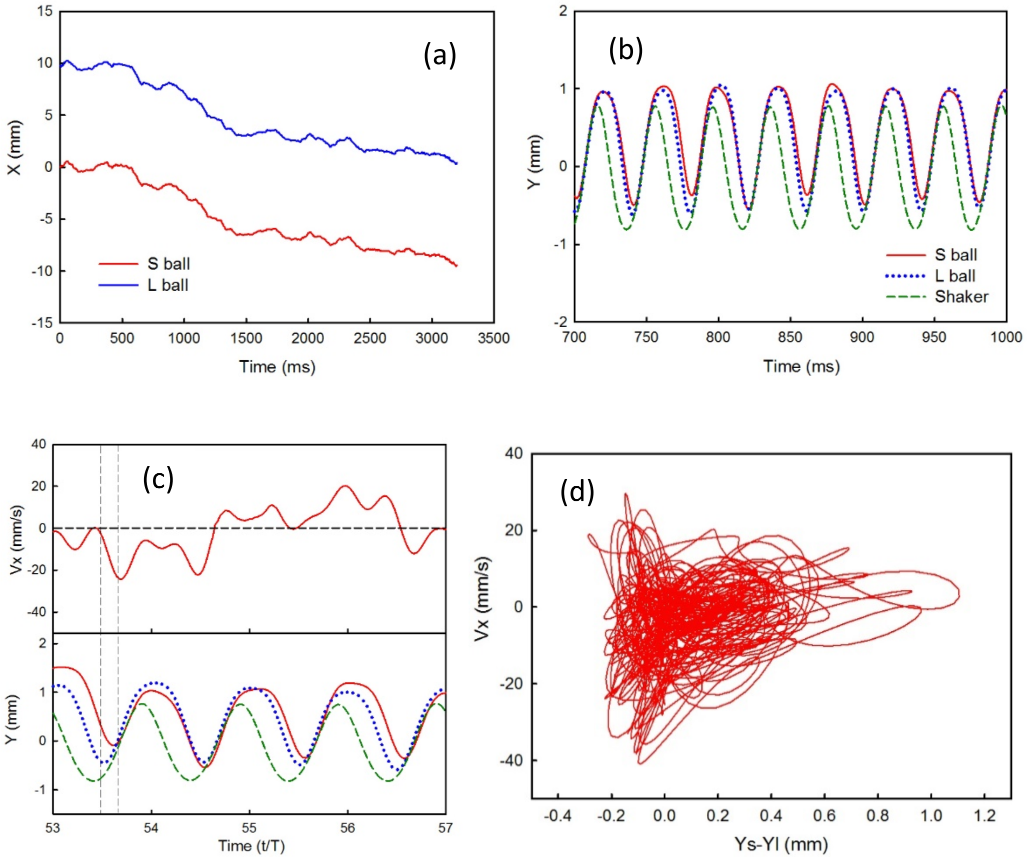

Fig.S8 TSB mode for a dimer with $\chi = 3.75$, $m = 19.35g$ ($\zeta = 3/8$), f=25 Hz, $\Gamma = 2.5$. (a) Horizontal position of the spherical centers of both ping-pong balls as a function of time. (b) Vertical position of the spherical centers of the both ping-pong balls and the vibrating plate as a function of time. (c) Upper panel, instantaneous horizontal velocity of S ball vs time in a typical TSB mode. Lower panel, vertical position of the both ping-pong balls and the vibrating plate as a function of time. Red solid curve: S ball; blue dotted curve: L ball; green dashed curve: shaker. The straight dashed line indicates the impact location. (d) Curves of horizontal velocity of S ball vs the vertical distance difference between the S and L ball for a complete measurement in (c), showing in an erratic manner.

Similar to the analysis in Fig.S3-5, the arbitrary choice of dimers together with the common kinetic features in Fig. S3-5 suggest the features are robust and the same underlying mechanism for all dimers dominate the TSB mode.

IV. Power density spectrum of velocities

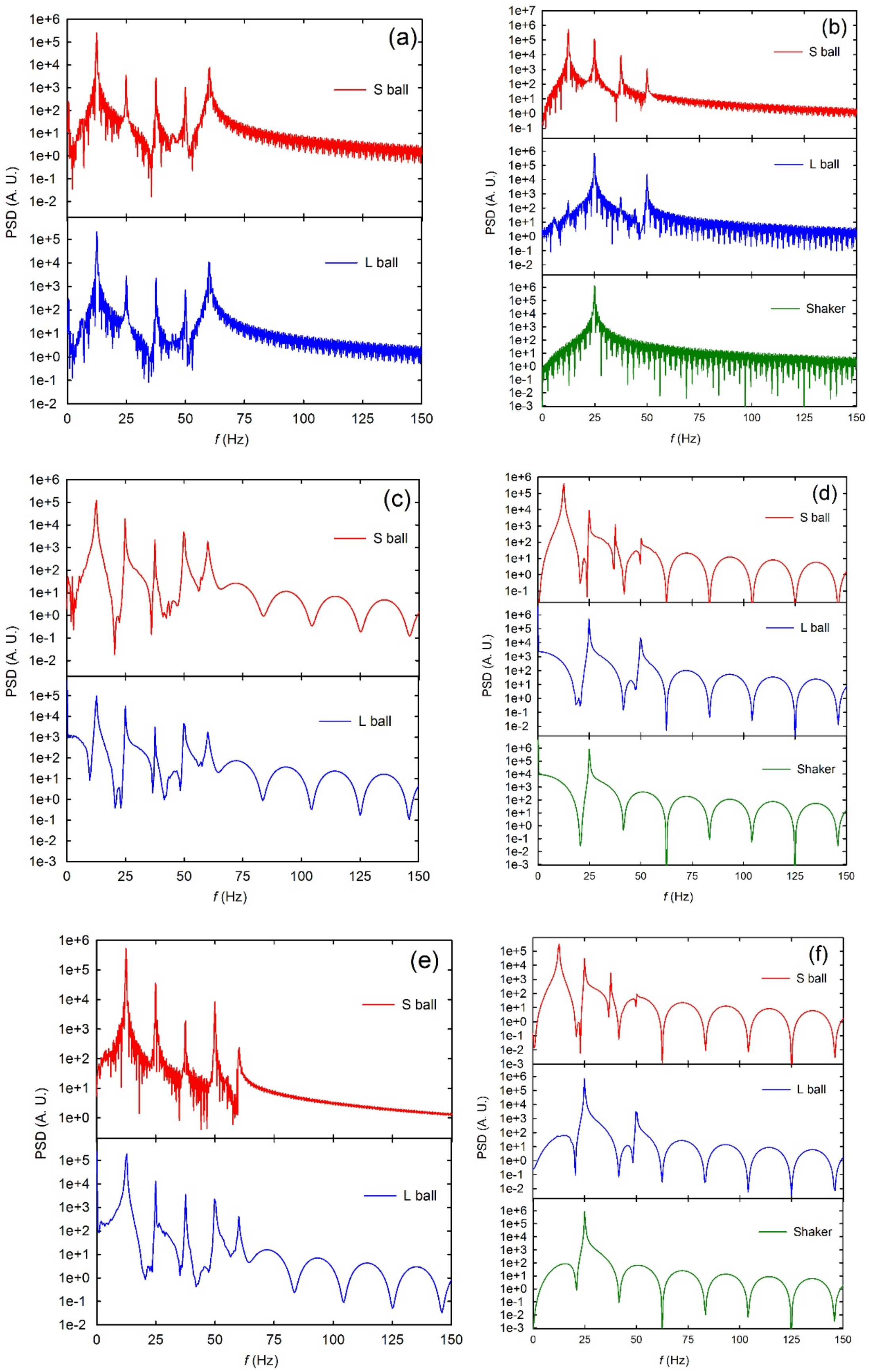

Fig.S9 Power density spectrum of the autocorrelation function of (a) Horizontal

velocity. (b) Vertical velocity. The parameters of the dimer in (a) and (b) are $\chi = 2.25$, $m = 12.91g$ ($\zeta = 1/4$), f=25 Hz, $\Gamma = 4.5$. (c) Horizontal velocity. (d) Vertical velocity. The parameters of the dimer in (c) and (d) are $\chi = 3.25$, $m = 12.91$ g ($\xi = 1/4$), f=25 Hz, $\Gamma = 4.5$. (e) Horizontal velocity. (f) Vertical velocity. The parameters of the dimer in (e) and (f) are $\chi = 3.75$, $m = 19.35g$ ($\xi = 1/4$), f=25 Hz, $\Gamma = 5$.

A power density spectrum (PDS) of the autocorrelation function of the velocity is an effective tool to analyze the dimer motion at x and y direction. Although the dimers are different in three cases above, it is surprising that their spectra seem very similar. It implies that the bouncing mode in TLB is stable. In particular, the main peak with largest height in all TLB cases shows at 12.5 Hz, exactly a half of the driven frequency of the shaker, confirms that the horizontal velocity undergoes a most important change in every two vibrating cycles.

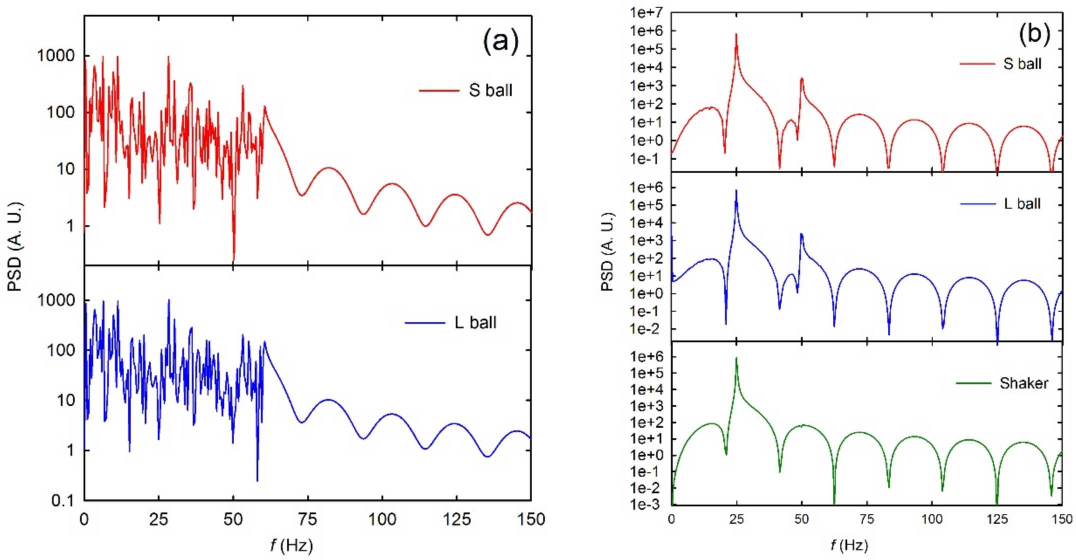

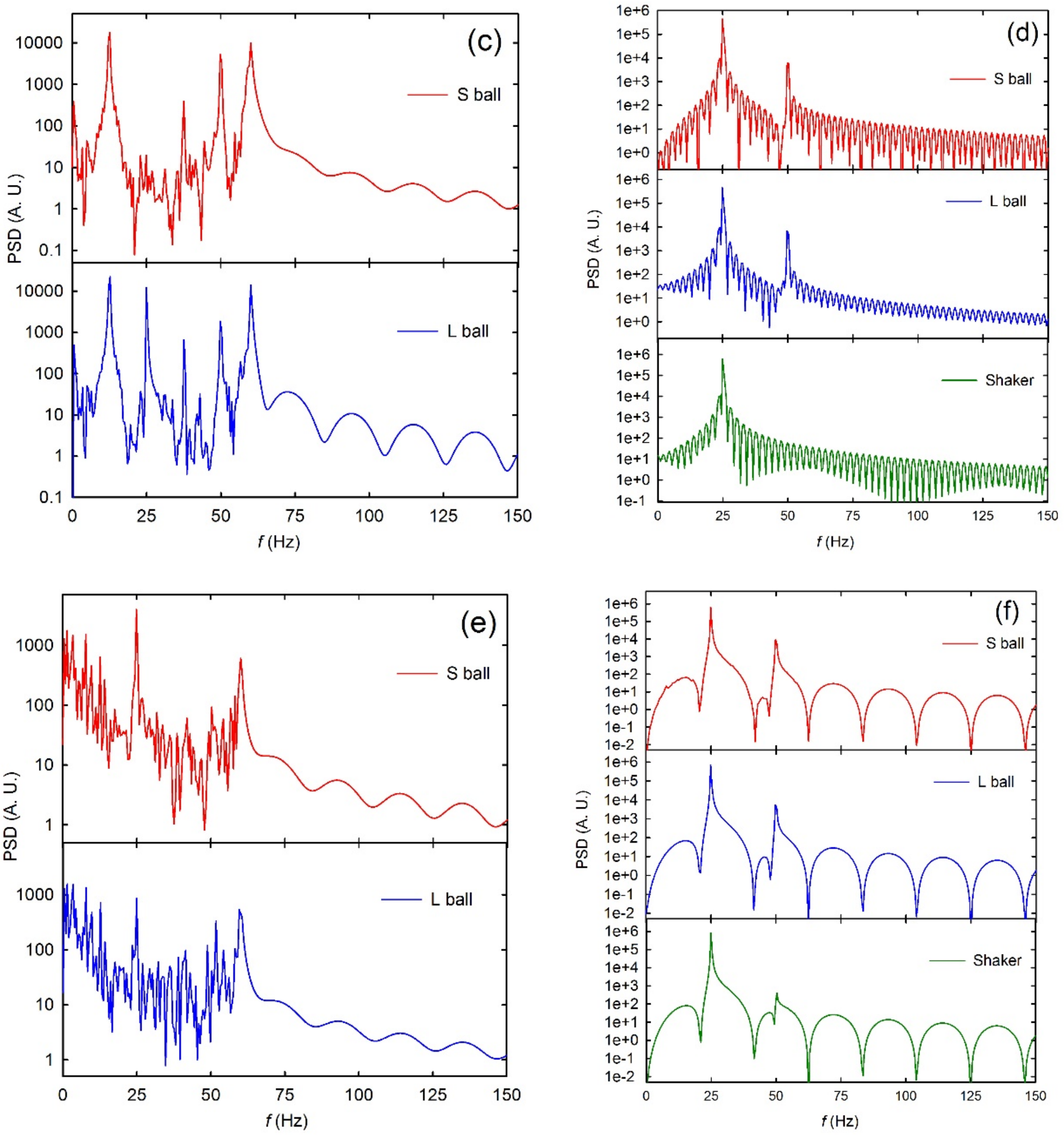

Fig.S10 Power density spectrum of the autocorrelation function of (a) Horizontal velocity. (b) Vertical velocity. The parameters of the dimer in (a) and (b) are $\chi = 2.25$, $m = 12.91g$ ($\zeta = 1/4$), *f*=25 Hz, $\Gamma = 2$. (c) Horizontal velocity. (d) Vertical velocity. The parameters of the dimer in (c) and (d) are $\chi = 3.25$, $m = 12.91$ g ($\xi = 1/4$), *f*=25 Hz, $\Gamma = 2$. (e) Horizontal velocity. (f) Vertical velocity. The parameters of the dimer in (e) and (f) are $\chi = 3.75$, $m = 19.35g$ ($\xi = 1/4$), *f*=25 Hz, $\Gamma = 2.5$.

Compare with the PDS in TLB mode, it is noteworthy that horizontal characteristic modes in TSB vary, depending on the specific conditions. The periodicity in horizontal velocity is not so obvious. The PDS in the vertical velocity is similar with the TLB mode, except that the frequency in the S ball is 25 Hz.

V. Time interval τ between two consecutive take-off

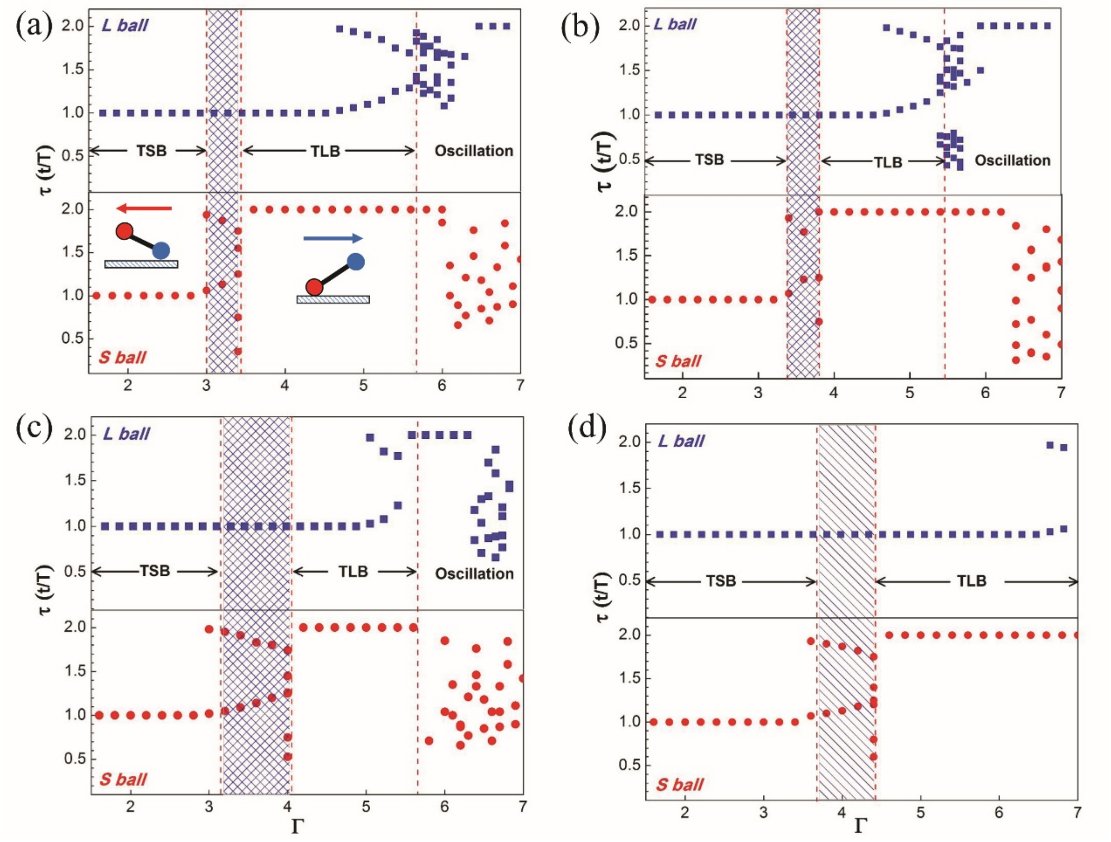

Fig.S11 (Color online) Time interval τ (normalized by the period T of the shaker) between two take-off locations on the vibrating plate is plotted as function of dimensionless acceleration Γ for different dimers. Upper (lower) panel indicates the L (S) ball. TSB and TLB regions are indicated by arrows, and the shadow area represents the crossover transition. (a) $\chi = 2.25$, $m = 6.455$ g ($\xi = 1/8$), f=25 Hz; (b) $\chi = 2.75$, $m = 12.91$ g ($\xi = 1/4$), f=25 Hz; (c) $\chi = 2.75$, $m = 25.82$ g ($\xi = 1/2$), f=25 Hz; (d) $\chi = 3.75$, $m = 12.91$ g ($\xi = 1/4$), f=25 Hz.

To depict the characteristic of the vertical bounce in both self-propelled modes, another important parameter τ, a time interval between two consecutive take-offs of a ball is presented in Fig. S11. In the TSB region, the time intervals τ of S or L ball are equal to one vibration period of the shaker ($\tau = T$), because the ball and plate stick together after a collision and the take-off location for each cycle is same. With increasing Γ to the shadowed area, there is bifurcation of τ for the S ball, which indicates that the vertical bouncing mode of the S ball varies. Within the shadowed

region τ remains the same for the L ball. The dimer used in the Fig. S11(b) and Fig. 2(a) is the same one; it is found that the width of the shadowed area on the Γ axis is consistent with that in the crossover region at 25Hz in the Fig. 2(a). This correspondence confirms that the shadowed area is the crossover region. After entering the TLB mode, τ for the L ball bifurcates, while τ doubles ($\tau = 2T$) for the S ball compared with the TSB mode. It is noteworthy that the discontinuous variation from T to $2T$ appears to be a critical signal to characterize the conversion between the TSB and TLB mode.

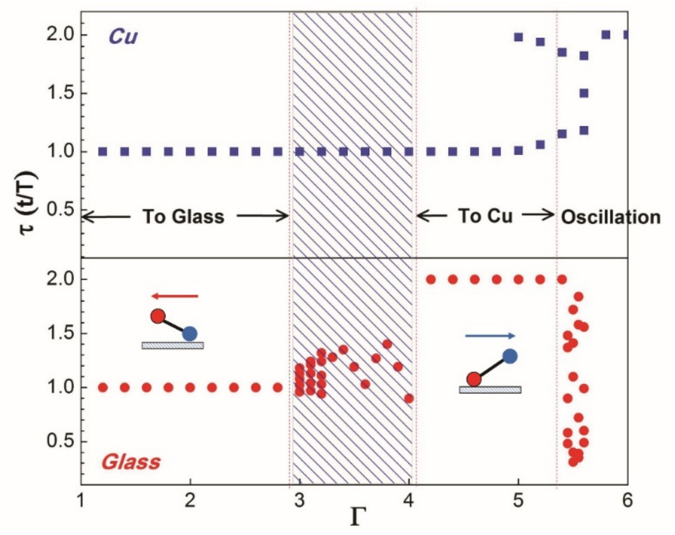

Fig.S12 Time interval τ (normalized by vibrating period T) vs the dimensionless acceleration Γ for a dimer filled with glass and copper spherical particles. The size of these particles is same, and the density is $\rho_{glass} = 2.5g/cm^3$ and $\rho_{cu} = 8.2g/cm^3$, respectively. Red (blue) ball in cartoon indicates the ball filled with glass (copper) particles. To glass (Cu) means that the self-propulsion is directed to the ball with glass (copper) particles, and the shadow area represents the transition region.

It is observed that the transition from the TSB to TLB mode is always associated with a discontinuous jump in τ. Interestingly, the discovery is valid for the dimer filled with different materials. We have used glass and copper particles with the same size and mass, and also found two types of directed self-propulsion: movement towards the glass-filled or copper-filled ball. The Fig.S12 shares a common structure with Fig.S11, namely there is a sharp transition when two directed motion shifts. The regularity in the time interval implies that the periodic bounce in the vertical direction

for both balls is a necessary condition for directed, self-propulsion. It seems the stochastic bouncing in both balls induces the oscillating state.

VI. Videos

Movie S1. The dimer exhibits a self-propelled, directed motion in a quasi-one-dimensional channel under vertical vibration. The dimer consists of two ping-pong balls connected by a rigid rod. Granular particles with the same mass and material are partially filled in both balls. However, the size of filling particles is different, diameter 0.3mm particles in one ball and diameter 3mm particles in another. $\Gamma = 2.5$, *f*=25Hz, $\chi = 2.25$ (rod length 10mm), $\xi = 1/4$ (filling mass 12.91g), and frames per second of this video (FPS) =100.

Movie S2. The particles filled in both balls are same (including size). $\Gamma = 4.5$, *f*=25Hz, $\chi = 2.25$ (rod length 10mm), $\xi = 1/4$ (filling mass 12.91g), and FPS=50.

Movie S3. Both balls are empty. $\Gamma = 2.5$, *f*=25Hz, $\chi = 2.25$ (rod length 10mm), $\xi = 0$ (filling mass 0g), and FPS=50.
